# Early retinal electrophysiology changes in quinine overdose

**DOI:** 10.1007/s10633-025-10034-1

**Published:** 2025-06-17

**Authors:** Stephanie Quinn, Vasileios T. Papastavrou, Clare Warriner, Jill M. O’Brien, Michael E. Grinton, Andrew C. Browning

**Affiliations:** 1 Visual Electrodiagnostics Service, Northern Medical Physics and Clinical Engineering, Newcastle upon Tyne Hospitals NHS Foundation Trust, Newcastle upon Tyne, UK; 2 Biosciences Institute, Newcastle University, Newcastle upon Tyne, UK; 3 Newcastle Eye Centre, Newcastle upon Tyne Hospitals NHS Foundation Trust, Newcastle upon Tyne, UK

**Keywords:** Electroretinogram, Quinine, Retinal toxicity, Inner retina, Electronegative, Connexin

## Abstract

**Purpose:**

To report the early and subsequent electrophysiological findings of 2 patients following quinine overdose.

**Methods:**

Serial assessments including: Medical history, visual acuity (VA), fundus autofluorescence, spectral-domain macular optical coherence tomography (OCT) and full-field electroretinogram (ffERG) were performed on 2 patients, between 2 and 47 days after quinine overdose.

**Results:**

Both patients experienced a similar clinical course. After almost total vision loss within the first 24 h, VA dramatically improved by day 3. Early OCT changes demonstrated central macula hyperautofluorescence, which coincided with a hyperreflectivity of the macular inner retina on OCT. The initial ffERG findings demonstrated changes consistent with marked inner retinal dysfunction of the cone system, affecting both the cone ON- and OFF-bipolar cell pathways. In contrast, rod bipolar cell function was unaffected in the early phase of toxicity. Between days 10 and 17, the retinal arterioles showed narrowing which coincided with attenuation of ffERG parameters of rod system inner retinal function between days 10–40.

**Conclusions:**

These cases suggest the early stages of quinine toxicity affect function of the presynaptic cone bipolar cell junction. This is then followed by retinal arteriolar attenuation and the well described electronegative scotopic ffERG.

## Introduction

In the UK, quinine can still be prescribed for both the treatment of malaria and nocturnal leg cramps, particularly those that disturb sleep. Therefore, patients remain susceptible to an inadvertent or deliberate overdose. Quinine toxicity can cause a wide range of signs and symptoms including visual disturbance [1]. Visual symptoms most often occur after a dose of 4 g while doses over 8 g are often fatal [2]. Typically, patients develop complete loss of vision between 10 and 24 h after taking an overdose; although central vision often improves over the next few days, peripheral visual loss remains [1, 3]. Debate remains over the cause of the visual symptoms.

This has led to suggestions that early changes in retinal function and vision are due to direct inner retinal toxicity, while the later changes are secondary to delayed retinal vascular attenuation [4–7]. Many reports [4, 5, 8, 9] describe the finding of an electronegative full-field electroretinogram (ffERG) in the late stages of quinine toxicity, however, very few descriptions of early ffERG changes have been reported. As quinine affects many ion channels and gap junctions within the body, including the retina, it is conceivable that the early effects of the drug on vision could be caused by the direct action of quinine on them [10–14]. Electrophysiology may therefore give insights into the early pathophysiological mechanism of quinine.

## Case 1

### Presentation

A 51-year-old man presented to the emergency department after taking an overdose of 4 g of quinine, 270 mg of mirtazepine and 12 g of ibuprofen 24 h earlier. After taking the overdose he had fallen asleep and on waking stated that he had complete loss of vision in both eyes. At presentation, vision was hand movements bilaterally and both pupils were dilated and unreactive to light.

### Days 2 to 3

**Fig. 1:**
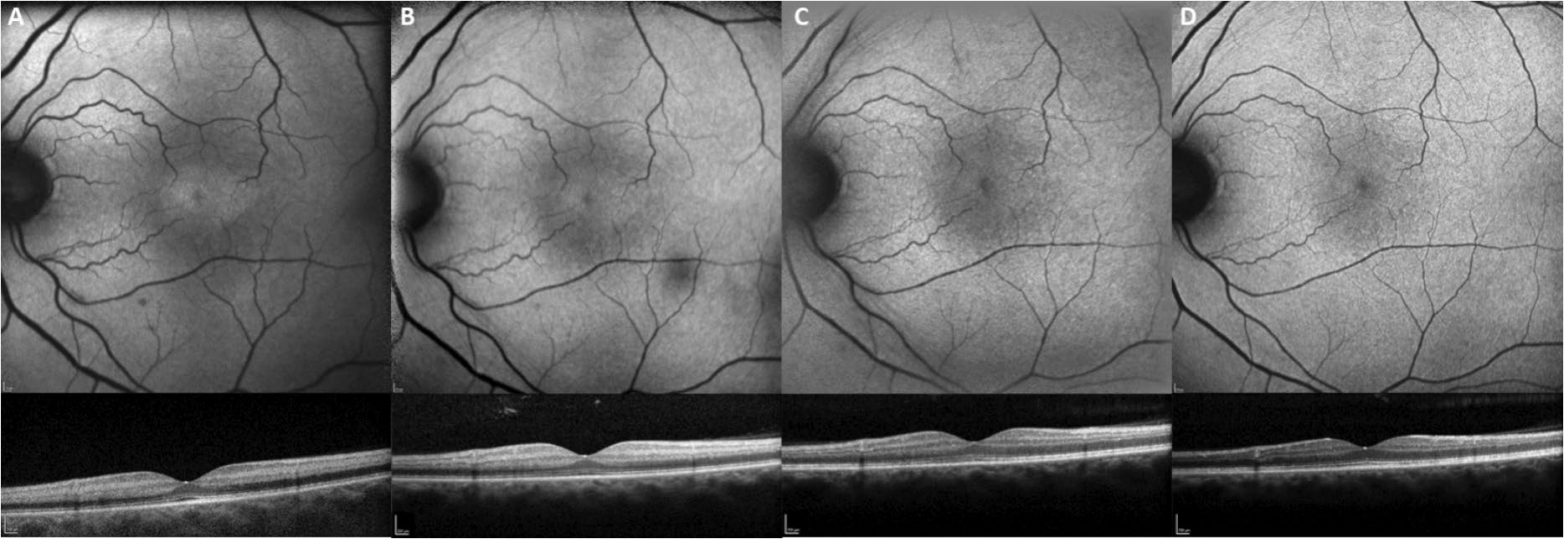
Heidelberg OCT and FAF images of the left eye of case 1 taken at day 2 (**a**), 5 (**b**), 11 (**c**) and 30 (**d**). The initial central macular hyperautofluorescence can be seen at day 2 (**a**), before gradually fading and becoming unnoticeable at day 30 (**d**). The OCT central macula scan shows hyperreflectivity of the inner retina from day 2–11, peaking at day 5 (**b**) and decreasing to day 30, with a suggestion of early inner retinal thinning (**d**)

The patient reported that his vision started to improve 2—3 days after the overdose. On examination, visual acuity (VA) was 6/6 OD (right eye) and 6/9 OS (left eye), and fundoscopy showed cloudy swelling of both maculae with marked inner retinal hyperreflectivity on macular OCT, similar to that found in the acute stages of a central retinal artery occlusion (Fig. 1a-b). Fundus autofluorescence (FAF) revealed bilateral central macular hyperautofluorescence (Fig. 1a-b), however a fluorescein angiogram showed normal retinal arteriolar calibre and flow, with no evidence of retinal ischaemia.

**Fig. 2:**
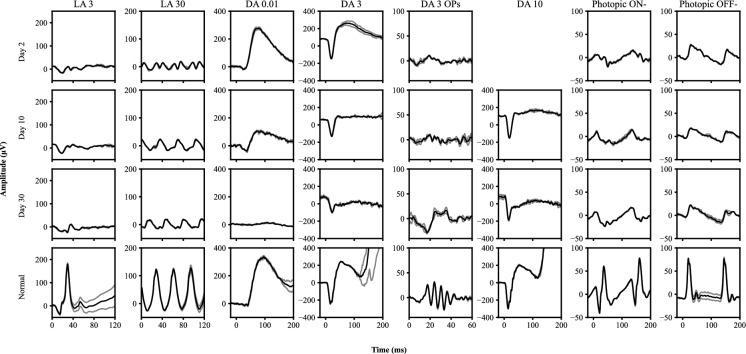
The full-field electroretinogram recorded from case 1 at day 2, 10 and 30, compared to a representative normal (age within 5 years of case). Only the left eye is presented as results from both eyes were largely symmetrical. The light intensity (cd·s/m^2^) is listed at header of the figure for each step and two individual results (grey) and an average of these results (black) are presented for each step. For the photopic responses each result has a minimum of 10 sweeps. The DA 10 response was not recorded at day 2 due to photophobia limiting the recording

The initial full-field electroretinogram (ffERG) was performed on day 2 using corneal gold foil electrodes (CH electronics) and Xenon flash (in house) or LED stimulator (Roland consult/Diagnosys) dependent on clinic/equipment availability (all calibrated to same standard) and followed the standards of the International Society of Electrophysiology of Vision (ISCEV) [15] (Fig. 2). Photopic ON–OFF responses were recorded using a previously described method [16]. Unfortunately, photophobia prevented the DA 10 response from being recorded, however, apart from oscillatory potential loss and on occasion mild peak time delays, other scotopic responses were within normal limits (Fig. 4 left panel, Table 1). The light adapted single flash (LA 3) responses demonstrated marked b-wave attenuation, while the light adapted 30 Hz flicker (LA 30) response was attenuated with a bifid/biphasic morphology. The LA 3 a-wave responses were only mildly subnormal and overall demonstrated a b:a ratio of 0.9 bilaterally implying cone system inner retinal dysfunction. The photopic ON–OFF ffERG showed attenuation of both ON- and OFF-responses.

**Table 1:** The recorded full-field electroretinogram parameters for case 1 and 2 at 3 similar time points. Highlighted bold cells in table indicate the results were outside of the reference limits, shown in lower row for each parameter (95th percentile)

Eye	Day	LA3 a		LA3 b		LA30 b		DA 0.01 b		DA3.0 a		DA3.0 b		DA 10 a		DA 10 b	
		µV	ms	µV	ms	µV	ms	µV	ms	µV	ms	µV	ms	µV	ms	µV	ms
*Case 1*																	
LE	2	**28.6**	**19.0**	**27.0**	31.0	**28.0**	**35.0**	293.6	74.0	227.0	**20.0**	407.6	**77.0**	-	-	-	-
LE	10	39.5	**18.0**	**37.0**	**39.0**	**36.7**	**36.0**	**143.3**	74.0	191.0	**21.0**	**234.1**	43.0	258.7	**19.0**	**288.6**	39.0
LE	30	**24.0**	**30.0**	**35.1**	**36.0**	**30.0**	**35.0**	**16.0**	**110.0**	**173.0**	**22.0**	**90.0**	37.0	274.0	**16.0**	**146.0**	39.0
RE	2	32.2	**18**	**36.3**	30	**31**	**35**	318.8	73	259.2	**19**	460.7	**78**	-	-	-	-
RE	10	40.3	**17.0**	**41.0**	**40.0**	**38.0**	**34.0**	**187.6**	79.0	231.4	**21.0**	**275.8**	42.0	285.7	**18.0**	**289.2**	39.0
RE	30	**17.6**	**24.0**	**27.6**	**35.0**	**29.3**	**35.0**	**31.0**	**103.0**	**187.0**	**21.0**	**111.0**	36.0	291.0	15.0	**140.0**	25.0
*Case 2*																	
LE	3	34.5	15.0	**71.6**	31.0	**75.2**	**32.0**	262.0	58.0	279.1	**19.0**	405.7	**72.0**	-	-	-	-
LE	7	44.6	**18.0**	**103.4**	**41.0**	**81.0**	**35.0**	317.1	78.0	205.2	**25.0**	497.7	**61.0**	-	-	-	-
LE	40	33.4	**18.0**	**44.6**	**38.0**	**47.8**	**34.0**	**158.3**	63.0	294.7	**21.0**	**278.5**	48.0	357.2	**16.0**	**276.8**	45.0
RE	3	38.7	16.0	**66.6**	31.0	**77.8**	**33.0**	344.6	58.0	308.1	**19.0**	500.5	**68.0**	-	-	-	-
RE	7	38.1	**17.0**	**94.8**	**41.0**	**78.8**	**36.0**	338.3	77.0	243.2	**23.0**	510.6	50.0	-	-	-	-
RE	40	40.2	**18.0**	**49.3**	**39.0**	**54.8**	**34.0**	**170.0**	63.0	289.1	**21.0**	**257.8**	**62.0**	353.4	**16.0**	**273.4**	47.0
*Reference limits*		31	16	142	32	86	31	193	101	190	18	380	60	229	15	394	60

### Days 5 to 10

The ffERG on day 10 demonstrated persistent LA 3.0 b-wave and LA 30 attenuation, now with additional peak-time delay but with resolution of the bifid LA 30 flicker morphology. The scotopic b-wave responses (DA 0.01 and 3) showed a reduction in amplitude of up to 51% from baseline and were now outside of reference limits. The DA 3 and 10 a-waves remained within reference range (Fig. 4). These findings coincided with the onset of retinal arteriolar attenuation.

### Day 30

At day 30, the VA was 6/6 OD and 6/7.5 OS and the inner retinal OCT hyperreflectivity had resolved. A Humphrey static threshold visual field test showed generalised constriction to within 10 degrees of fixation bilaterally. The photopic ffERG responses demonstrated persistent LA 3 b-wave and LA 30 attenuation, with additional mild attenuation of LA 3 a-waves. The scotopic ffERG showed further attenuation, affecting the DA 0.01, DA 3 and DA 10 b-waves. Additional a-wave attenuation was seen in the DA 3 response, up to a 28% reduction from baseline, now sitting outside of the lower reference limit bilaterally (Fig. 4). The DA 10 a-waves remained within the reference range, resulting in a truly electronegative waveform with a b:a ratio of 0.48 and 0.53 in the right and left eye, respectively.

## Case 2

### Presentation

A 45-year-old female presented to the emergency department after taking an overdose of quinine (4.8 g) along with small amounts of domperidone, co-codamol, sumatriptan, pizotifen, duloxetine and rabeprazole the previous day. In the first 18 h after quinine overdose, she developed complete loss of vision in both eyes. She reported that the central vision started to improve on the second day after the overdose.

### Days 2 to 4

**Fig. 3:**
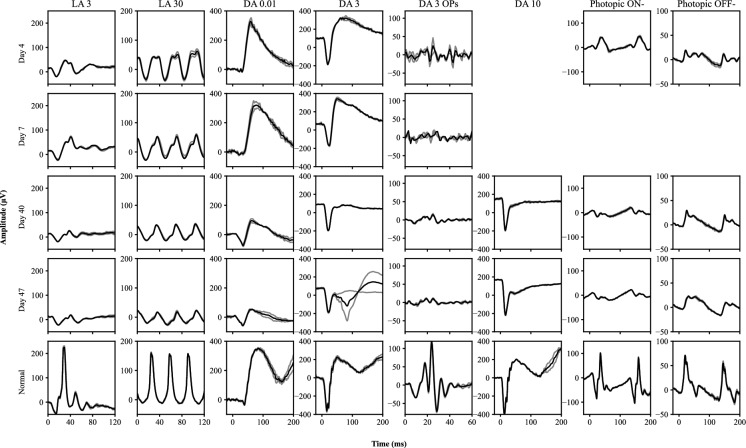
The full-field electroretinogram recorded from case 2 at day 4, 7, 40 and 47, compared to a representative normal (age within 5 years of case). Only the right eye is presented as results from both eyes were largely symmetrical. The light intensity (cd·s/m^2^) is listed at header of the figure for each step and two individual results (grey) and an average of these results (black) are presented for each step. For the photopic responses each result has a minimum of 10 sweeps. The DA 10 was not recorded at day 4 and 7 and the photopic ON–OFF ffERG was not recorded at day 7 due to photophobia limiting the recording

**Fig. 4:**
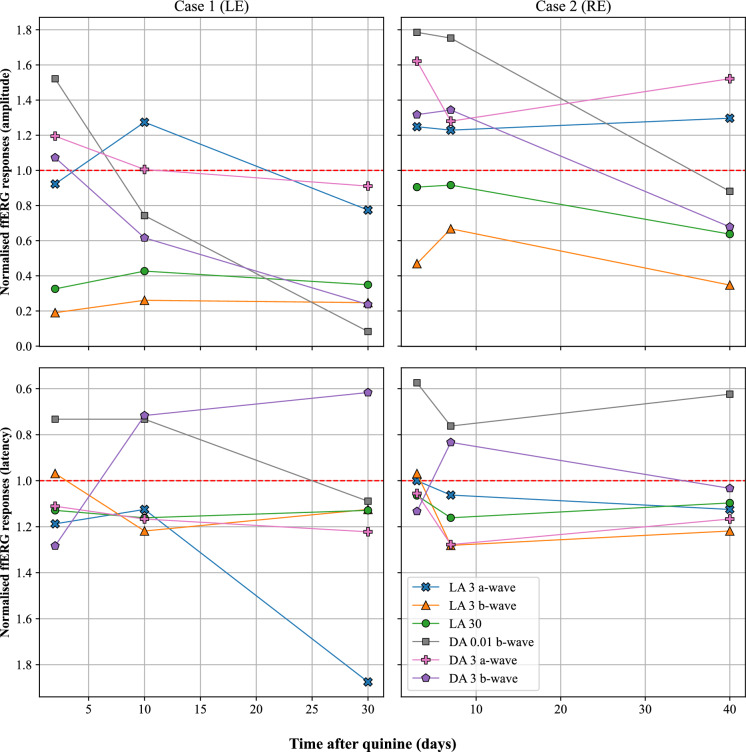
The full-field electroretinogram data normalised to the upper reference limit (95th percentile) for case 1 (represented by left eye data) and case 2 (represented by right eye data), for both amplitude (upper panels) and implicit time (lower panels), plotted against the days after quinine overdose. The reference limit is indicated by the red dashed line. The DA 10 response was not available at every time point and therefore not included. The y-axis for the latency data is inverted

On day 3, VA was 6/5 both eyes and fundoscopy showed opaque swelling of both maculae, with inner retinal hyperreflectivity on OCT and central macular hyperautofluorescence on FAF. Similar to case 1, the ffERG recorded on day 4 demonstrated normal scotopic retinal function, with the exception of poorly formed oscillatory potentials (Fig. 3). There was marked attenuation and delay of the LA 3 b-wave and the LA 30 responses (Fig. 4). In addition, the LA 30 response demonstrated a distinctive bifid peak similar to case 1. The photopic ON–OFF ffERG responses were consistent with both ON- and OFF- cone pathway dysfunction (Fig. 3).

### Day 7 to 17

The ffERG recorded on day 7 was largely comparable to the recording on day 4, with further worsening of oscillatory potentials (Fig. 3). On day 11 VA was 6/9 both eyes, the macular OCT scans showed attenuation of the inner retinal hyperreflectivity and the central macular hyperautofluorescence had resolved. A Goldman visual field examination showed generalised concentric constriction to approximately 15 degrees of fixation. On day 17, some mild retinal arteriolar attenuation was noted.

### Day 40 to 47

Subsequent ffERGs recorded on day 40 and 47 demonstrated mild worsening of LA 3 b-waves (b:a ratio approaching 1) and the LA 30 response (Fig. 3). The scotopic ffERG demonstrated marked loss of b-waves in the DA 0.01, DA 3 and DA 10 responses however the a-waves remained unaffected, resulting in electronegative DA 3 and DA 10 ffERGs.

## Discussion

Since Smith reported “the paradox of quinine phenomenon” in 1919 [17], the effect of quinine toxicity on the retina has remained poorly understood. Using retinal electrophysiology, early researchers suggested that the initial visual manifestations of quinine toxicity were caused by direct effects of the drug on the retina, before the well-recognised, delayed retinal arteriolar narrowing and electronegative ffERG developed [4, 7, 9]. Unfortunately, the early changes and effects on the human retina are limited to reports that are confined to non-ISCEV compliant, predominantly scotopic responses and often appear contradictory in terms of the effect of the drug on the retina with some authors reporting inner and outer retinal dysfunction [3, 4, 18] while others suggest normal retinal function [6, 7].

In this study we describe the novel early electrophysiology findings in 2 cases of acute quinine toxicity that suggest the initial (days 2–7) pathological effects of quinine are predominantly at the level of the cone inner retina. The early ffERGs in both cases showed signs of dysfunction primarily in the inner retina of the cone system, with loss of the LA 3 b-wave and an attenuated and bifid LA 30 response (Fig. 4). In addition, specific testing also demonstrated that both the ON- and OFF- pathways were affected (Figs. 2 and 3). Interestingly, the duration of selective cone system inner retinal dysfunction appears to coincide with the time that quinine is predicted to remain detectable in the blood after quinine overdose [19]. Rod system inner retinal function (DA 0.01) appeared well preserved in the early stages of toxicity (up to day 7 post overdose). These photopic ffERG findings are similar to those seen in inherited retinal dystrophies characterised by a loss of cone photoreceptor to bipolar cell transmission such as incomplete congenital stationary night blindness [20] associated with mutations in CACNA1F [21] and other cone-rod synaptic disorders caused by mutations in CABP4 [22] and CACNA2D4 [23]. These conditions are associated with abnormal function of photoreceptor presynaptic voltage dependent calcium channels, therefore suggesting a similar early effect of quinine on these ion channels. Similar to these conditions in both cases the bipolar OFF-pathway d-wave responses show an abnormal morphology which has been reported previously in later stages of toxicity [8]. This further supports the initial site of action of quinine toxicity being at the level of the cone photoreceptor to bipolar cell synapse. To the best of our knowledge, our cases are the first to show these early cone system inner retinal changes prior to the involvement of the rod system. Both cases later develop rod system involvement with marked loss of scotopic b-wave amplitude and electronegative waveforms developing between days 10 and 40, consistent with previous reports [4, 5, 8, 9]. The scotopic ffERG changes appear to parallel the development of the widely reported finding of retinal arteriolar attenuation.

Quinine is known to have a number of effects on mammalian cells, from blocking sodium and potassium channels, to an antagonistic action on acetylcholine receptors [14, 24]. There is little evidence however, that it directly affects calcium channels. Interestingly, it has been shown to block gap junction channels containing Connexin-36 (the most abundant connexin in the mammalian retina) and Connexin-50 [13, 25]. Connexin-36 is found on both rod and cone photoreceptors, cone bipolar cells and AII-amacrine cells and is thought to be important in the transmission of signals through the retina [26, 27]. Recently Jiang et al., found that myopic patients with changes at a gene locus close to the GJD2 gene (which encodes Connexin-36) demonstrated alterations in cone driven OFF- responses [28]. These changes have similarities to our cases and would suggest that some of the early ffERG changes in quinine toxicity may be due to the blocking of Connexin-36 associated gap junction currents in the cone system, which could be necessary for the transmission of the signal from the cone photoreceptors to bipolar cells. This is supported by the early loss of the photopic b-wave in quinine toxicity, whereas, the scotopic b-wave is initially preserved and Connexin-36 is not known to be expressed on the rod bipolar cells. In summary, these findings suggest that in cases of quinine toxicity, cone photoreceptor to bipolar cell transmission is affected early in the course of the disorder, possibly by a direct action of the drug and that this may be mediated in part by the effect of quinine on Connexin-36 gap junction blockade. This is followed later by retinal vessel narrowing and the concomitant loss of scotopic inner retinal function. Photoreceptor function appears at worst, only mildly affected throughout.
